# An Entropy-Based Position Projection Algorithm for Motif Discovery

**DOI:** 10.1155/2016/9127474

**Published:** 2016-11-02

**Authors:** Yipu Zhang, Ping Wang, Maode Yan

**Affiliations:** Department of Automation, School of Electronics and Control Engineering, Chang'An University, Xi'an 710064, China

## Abstract

Motif discovery problem is crucial for understanding the structure and function of gene expression. Over the past decades, many attempts using consensus and probability training model for motif finding are successful. However, the most existing motif discovery algorithms are still time-consuming or easily trapped in a local optimum. To overcome these shortcomings, in this paper, we propose an entropy-based position projection algorithm, called EPP, which designs a projection process to divide the dataset and explores the best local optimal solution. The experimental results on real DNA sequences, Tompa data, and ChIP-seq data show that EPP is advantageous in dealing with the motif discovery problem and outperforms current widely used algorithms.

## 1. Introduction

Motif discovery problem is an issue of discovering short similar nucleotide segments with a common biological function, which is crucial for understanding the structure and function of gene expression. Quickly and accurately locating motif is a challenging problem in computational biology.

A challenge of motif discovery problem is described as follows [1]: find a motif of length *l* in *t* gene sequences. Each sequence is *n* nucleotides long and contains one motif instance with up to* d* mutations to the true motif.

Over the past decades, numerous algorithms have been proposed to identify motifs in several to dozens of promoter sequences from coregulated or homologous genes [2]. These algorithms can be divided into two categories: One is exact algorithms, which use consensus sequences to represent motifs [3]. Recent exact algorithms mainly concentrate on pattern-driven algorithms [4–8]. They scan all sequence patterns of length *l* with an initial search space of O(4^*l*^) and report all possible solves. These pattern-driven based algorithms are able to deal with larger amount of sequences like ChIP-seq data [2, 9]. However, they are exponential-time algorithms that need a great deal of time to search for the larger* l* and inefficient for handling dozens of sequences.

The other category is approximate algorithms, which use the position weight matrixes (PWMs) to represent motifs [10]. The approximate algorithms commonly establish probability training model and score a statistical measure to identify biological signals from background. A particularly successful class of approximate algorithms is developed based on Gibbs sampling [11] and MEME [12]. MEME finds motifs by optimizing the PWMs using the Expectation Maximization (EM), which still defines three types of motif discovery sequence model: OOPS, ZOOPS, and TCM, corresponding to one occurrence per sequence, zero or one occurrence per sequence, and zero or more occurrences per sequence, respectively. The probability training algorithms have been widely used due to its simplicity and stability. The primary advantage of approximate algorithms is the speedy runtime and minimal memory consumption. Random Projection [13] is a projection-based approximate algorithm which projects all substrings of length *l* into the buckets by hashing and then derives the consensus sequences to select some valid buckets. VINE [14] is a graph-based motif discovery algorithm which finds motif by clustering cliques in a *t*-graph. APMotif [15] applies Affinity Propagation to cluster and then employs an effective EM refinement to search for optimal motifs. However, the performances of these algorithms strongly depend on the starting positions, which cause the convergence easy to fall into local optimum, and the training iteration executes much slower when the width of motif increases in the larger data.

In order to overcome these shortcomings, in this paper, we propose an entropy-based position projection algorithm for motif discovery, named EPP. We design a projection method to divide the dataset into candidate subsets by utilizing the relative entropy in each position of motif. Then, EPP filters the candidate subsets and refines the subsets by searching all the possible instances. We consider intramotif dependency in statistics model and calculate the average log-likelihood ratio to combine the short motif. Our algorithm can apply to OOPS, ZOOPS, and TCM sequence model through the threshold setting. Experimental results on real DNA sequences, Tompa data, and ChIP-seq data demonstrate that EPP is advantageous to deal with the motif discovery problem and outperforms current widely used approximate algorithms.

## 2. Materials and Methods

### 2.1. Notations

Given an input set of sequences **S** = {*S*_*i*_∣*i* = 1, 2, 3, …, *t*} over the alphabet Σ, the length of sequence *S*_*i*_ is *n*_*i*_, the length of the motif to be discovered is *l*, and the number of mutations allowed is *d*. The substring, *x*_*ij*_ = (*s*_*ij*_, *s*_*i*,*j*+1_, …, *s*_*i*,*j*+*l*−1_), starting at position *j* of the *i*th sequence is defined as an *l*-mer. For sequence *s*_*i*_, there are *n*_*i*−*l*+1_ substrings of length *l*. Let set **X** be the set of all the substrings of **S**. *q* is the projection position. Here, |Σ| = 4 for DNA datasets and |Σ| = 20 for the protein sets.

### 2.2. Motif Representation

**Figure 1:**
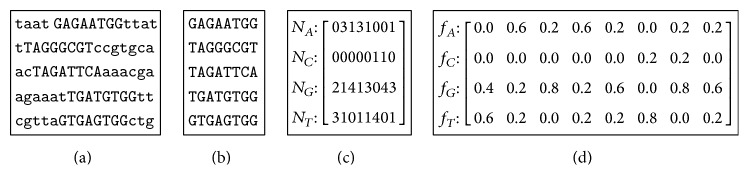
The process of calculating the PFM. (a) The input sequences. (b) The aligned substrings. (c) The count matrix. (d) The position frequency matrix.

Generally, a motif can be drawn from a multinomial distribution [16], *F* = (*f*_1*k*_, …, *f*_*wk*_, …, *f*_*lk*_)  (*k* ∈ Σ), where *f*_*wk*_ represents the probability of nucleotide *k* preference at the *w*th position of the motif and *f*_0*k*_ represents the background probability of nucleotide *k*. The position frequency matrix (PFM) **F** can be obtained by calculating the frequency of each nucleotide *k*  (*k* ∈ Σ) at each aligned site:

(1) $$\begin{array}{c} f_{wk}=\frac{N_{wk}+\varepsilon }{\sum_{k\in \Sigma }N_{wk}+4\varepsilon }, \end{array}$$

where *N*_*wk*_ is the count of an observed nucleotide *k* at position *w* and  *ɛ* indicates the pseudocounts to deal with the zero frequencies.  Figure 1 describes how to calculate the PFM through the input sequences.

Information content (IC) is a measure to rank the motif conservation [17]. Motifs with higher IC represent they have more specific binding preferences. Suppose we have a motif built from the PFM of the selected substrings; the information content of the *w*th position of the motif is defined as

(2) $$\begin{array}{c} I_{w}=\underset{k\in \Sigma }{\sum }f_{wk}\log\left(\;\frac{f_{wk}}{f_{0k}}\;\right). \end{array}$$

Due to the independence of the positions of the motif, the information content of motif is

(3) $$\begin{array}{c} I=\overset{l}{\underset{w=1}{\sum }}I_{w}. \end{array}$$

The IC can be used to rank motifs with the same length* l*. However, some researches indicate that the commonly multinomial distribution model may be too simplistic in identifying the binding motifs, while some positions of TF binding motif exert an interdependent effect on binding affinities of TFs [18, 19]. To provide a better result of motifs identification, a more sophisticated model that involves the intramotif dependency should be considered. Intramotif dependency considers that the frequency of nucleotide combinations spanning several positions deviates from the expected frequency under the independent motif distribution [20]. For example, if the frequency of two nucleotides, “GT,” in a pair of positions is much higher or lower than the product of frequency of “G” in the first position and the frequency of “T” in the second position, we infer that these two positions are dependent. Therefore, the log-likelihood of nucleotides *s*_*i*_ and *s*_*i*+1_ is

(4) $$\begin{array}{c} p\left(\;s_{i},s_{i+1}\;\right)=log\frac{\Phi_{i,i+1}\left(s_{i},s_{i+1}\right)}{\Phi_{0}\left(s_{i},s_{i+1}\right)}, \end{array}$$

where Φ_*i*,*i*+1_ represents the probability of the nucleotide pair at *i*th and (*i* + 1)th position of the motif and Φ_0_ represents the background probability of the nucleotide pair. Then, the conditional probability of the substring *x* is

(5) px ∣ F=log∑w=1l∑k∈Σfwk·∑w=1l−1∑k1,k2∈ΣΦw,w+1k1,k2p0x,

where *p*_0_(*x*_*ij*_) is the joint probability under the corresponding background distribution *f*_0_. In this paper, we use the third-order Markov model to characterize the background sequence and improve the sensitivity and specificity of identifying motifs. The probability of the substring *x*  (*s*_*ij*_, *s*_*i*,*j*+1_, …, *s*_*i*,*j*+*l*−1_) in the background under a third-order Markov model is

(6) p0xpsipsi+1 ∣ sipsi+2 ∣ si,si+1⋯psi+l−1 ∣ si+l−2,si+l−3,si+l−4.

So the information content can be represented as

(7) $$\begin{array}{c} I=avg\sum p\left(\;x\;|\;F\;\right)log\left(\;\frac{p\left(x\;|\;F\right)}{p_{0}\left(x\right)}\;\right). \end{array}$$

Based on the substring statistical significance representation, we present a novel entropy-based position projection algorithm (EPP). EPP aims to solve the motif identification problem and make a good trade-off between accuracy and efficiency, which is detailedly described as follows.

*EPP Algorithm*

**Step 1 (the cluster projection process).**

Since the random initial state contains too much noise information, how to choose a good initial state to make refinement quickly converge to a local optimal solution becomes essential. Obviously, the (*n*_*i*_ − *l* + 1)^*t*^ ways of selecting the* l*-mers from all substrings to constitute the initial state are too large. Here, we designed a cluster projection method to initialize the parameters:

(1) Draw all the substrings from dataset **S** to form a new set **X**,  **X** = {*x*_*n*_∣*n* = ∑(*n*_*i*_ − *l* + 1)}, where *x*_*n*_ represents an *l*-mer.

(2) Calculate the relative entropy of each position in the set** X**:

(8) $$\begin{array}{c} H_{w}=\underset{k\in \Sigma }{\sum }f_{wk}\log\left(\;\frac{f_{wk}}{f_{0k}}\;\right),\;\left(w=1,\ldots ,l\right). \end{array}$$

(3) Select the position *q* of the maximum relative entropy as the projection position, *q* = argmax_*w*=1,…,* l*_⁡{*H*_*w*_}. The collection set **X** is divided into four subsets through the projection process: the first subset *X*_1_ contains all the *l*-mers of appearing base “A” in position *q*. Similarly, the subsets *X*_2_,  *X*_3_, and *X*_4_ contain all the *l*-mers of appearing bases “C,” “G,” and “T” in position *q*, respectively.

(4) We set two thresholds max_size and min_size to check the size of the subsets {*X*_1_, *X*_2_, *X*_3_, *X*_4_}. For example, if |*X*_1_| < min_size, we abandon *X*_1_. That is, *X*_1_ is too small to contain enough motif instances, which means a transcription factor cannot be combined with sufficient sequences; if |*X*_1_| > max_size, the subset has much unnecessary background noise, the algorithm should be back to (2), and we find a new projection position to further divide *X*_1_; if min_size ≤ |*X*_1_| ≤ max_size, we consider *X*_1_ is qualified and store it into a candidate set {*c*_*m*_}. The setting of max_size and min_size will be described in next section.

**Figure 2:**
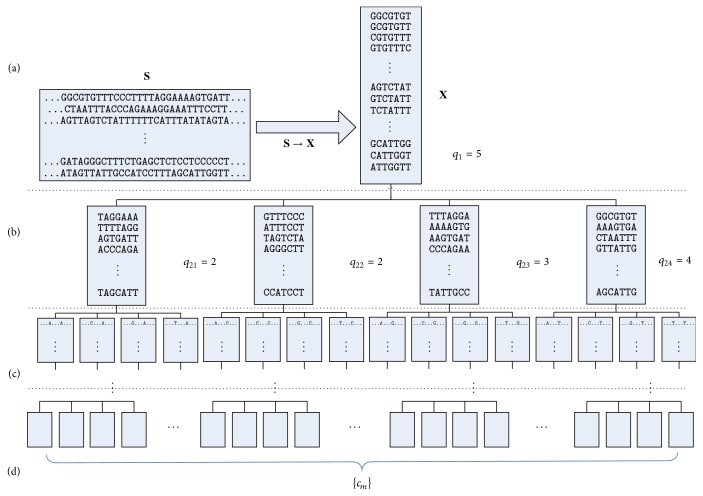
The process of cluster projection.

Figure 2 shows an example of the cluster projection process. Figure 2(a) describes the set** X** derived from** S**; we choose the fifth position for projection. Figure 2(b) shows the four subsets divided from** X**; the fifth positon of each subset is the observing letters “A,” “C,” “G,” and “T,” respectively. Then, we calculated relative entropy and chose the second, the third, and the fourth position of each subset to project. After several projection processes (Figure 2(c)), we obtain a candidate set {*c*_*m*_} as shown in Figure 2(d).

In the worst case, the maximum number of candidate subsets is *n*/min_size *n* is the number of all substrings (*l*-mer). However, in practice, the number of candidate subsets will be much less than this number, such that when the number of substrings is 10^5^, the number of candidate subsets is ultimately only a few hundred.

**Step 2 (filter the candidate set).**

The candidate set {*c*_*m*_} is constituted by a series of cluster subsets which form by the similar substrings of the same letters at several positons. However, the candidate set still contains the useless subsets made up by the background. It will cost a lot to refine these background subsets and it is necessary to filter them.

Because the projection process calculates the relative entropy to choose the position, it can measure the statistical significance but cannot reflect the complexity of substrings. In order to evaluate the complexity of each subset, we employ the common single-string score [21] as another measure.

(9) $$\begin{array}{c} J\left(\;m\;\right)={\left(\;\frac{1}{4}\;\right)}^{l}\underset{k\in \Sigma }{\prod }{\left(\;\frac{l}{\sum_{w=1}^{l}f_{wk}}\;\right)}^{\sum_{w=1}^{l}f_{wk}}. \end{array}$$

So we filter each subset of {*c*_*m*_} by computing the complexity function (9) and the content information (7) as follows:

(1) Calculate the complexity score of each subset in {*c*_*m*_}, denoted by *J*(*m*):

(10) $$\begin{array}{c} \bar{J\left(m\right)}=\frac{1}{\left|m\right|}\sum J\left(\;m\;\right), \end{array}$$

where |*m*| represents the cardinality of {*c*_*m*_} and *φ*_*J*_ represents the radius of complexity.

(11) φJ=maxmaxJm−Jm¯,minJm−Jm¯.

(2) Calculate the content information of each class in {*c*_*m*_}, denoted by *I*(*m*):

(12) $$\begin{array}{c} \bar{I\left(m\right)}=\frac{1}{\left|m\right|}\sum I\left(\;m\;\right). \end{array}$$

Similarly, let *φ*_IC_ be the radius of IC:

(13) φIC=maxmaxIm−Im¯,minIm−Im¯.

(3) For each candidate subset in {*c*_*m*_}, if it satisfies

(14) $$\begin{array}{c} \left|\;J\left(\;m\;\right)-\bar{J\left(m\right)}\;\right|>\varphi_{J}\;\&\&\;\left|\;I\left(\;m\;\right)-\bar{I\left(m\right)}\;\right|>\varphi_{IC}, \end{array}$$

this subset is considered qualified and saved into **G** = {*G*_*v*_}.

**Step 3 (refine the qualified subsets).**

Assume each qualified subset *G*_*v*_ corresponds to a motif; the substrings of the qualified subset should be the motif instances. In fact, we found that the qualified subset contains several fake motif instances generated by the background sequences, while some instances may be missed by the projection and filter processes and are not in the qualified subsets. Therefore, in this step, we remove the fake instances and add the missing ones to refine each qualified subset.

As the previous study [22], we know the instances *M*_1_ and *M*_2_ of the same motif should be satisfied *D*_*H*_(*M*_1_, *M*_2_) ≤ 2*d*, where *D*_*H*_(·) is the function of measuring the hamming distance between two substrings. For each qualified subset in *G*_*v*_, if the substring of the qualified subset satisfies the hamming distance less than or equal to 2*d* from the others, we keep it in the subset; otherwise, we remove it from the subset. For each fixed *l*, the value of *d* is usually set as *d* < *l* = 2. In this way, the real motif instances must be in one qualified subset.

Then, we search all the possible instances from **X** and add them into *G*_*v*_. The possible instances should satisfy the following two conditions. First, the instance *x* satisfies

(15) $$\begin{array}{c} D_{H}\left(\;x,\frac{1}{\left|G_{v}\right|}\underset{g\in G_{v}}{\sum }g\;\right)\leq 2d, \end{array}$$

where |*G*_*v*_| is the cardinality of *G*_*v*_ and  *g* represents one instance in *G*_*v*_. Second, adding the instance *x* increases the information content (7) of *G*_*v*_. These limiting conditions greatly reduce the search space, and we can obtain the refinements for each qualified subset after removing and adding the substrings. In addition, if the qualified subset is too small (less than min_size), it indeed does not make sense to contain the real motif instances. We will not refine the small qualified subset and drop it.

**Step 4 (predict the longer motif).**

See each qualified subset as a seed, its PWM can be computed by the steps above, while the corresponding motif with high information content can also be calculated. However, the qualified subsets may represent the similar motifs with a few letters varying as previous studies [23, 24]. In order to eliminate redundant motif information and expand the short motif to form longer motif, we combine the similar motifs having the long common-overlap segments by utilizing a metric of computing the average log-likelihood ratio (ALLR) [25]:

(16) ALLRx1w1,x2w2=∑kNw2kln⁡fw1k/f0k+∑kNw1kln⁡fw2k/f0k∑kNw1k+Nw2k,

where *f*_0*k*_ is the background frequency of base *k* and *N*_*w*_1_*k*_/*N*_*w*_2_*k*_ and *f*_*w*_1_*k*_/*f*_*w*_2_*k*_ are the count and frequency of base *k* at the *w*_1_th/*w*_2_th position of *x*_1_/*x*_2_. Since the length of predicted motifs may be different, we use the minimum distance between motifs among all possible overlaps of motifs *x*_1_ and *x*_2_ that the aligned segment is 6. Thus, we calculate the similarity score of *x*_1_ and *x*_2_ by (17), where *l*_*s*_ denotes the length of the segment:

(17) simx1,x2=maxw1,w2∑hls−1ALLRx1w1+h,x2w2+h.

Suppose the number of motifs to find is *u*; when a new motif is found, we first check whether there is a similar motif. If the similar motif exists, we combine them and obtain the longer motif; if the similar motif does not exist, we keep the new motif and replace the motif with minimum information content. In this way, we ensure the *u* motifs are different which are also have the information contents as high as possible. In practice, we finally combine and generate at least 20 top information content motifs as the outputs.

The whole algorithm of EPP is described in Algorithm 1.

In Step 1, lines (1) to (14), we make the projections to obtain candidate sets; then lines (15) to (17) are the step to filter candidate sets to get the qualified subsets; lines (18) to (23) are the step to refine each qualified subset; at last, lines (24) to (26) are the step to combine the similar motifs and output the results.

## 3. Results and Discussion

The parameters we can get from the input dataset include the number of sequences *t* and the length of each sequence *n*_*i*_  (*i* = 1, …, *t*); the motif length *l* is known (6–30 bps). Based on these parameters, we draw the set **X** and then start the projection process. The times of projection and the number of the candidate subsets are depending on the parameters of max_size and min_size. We hope that the candidate subsets containing the true motif have the motif instances as more as possible and have less influence by the background. Thus, for different sequence models, the parameters of max_size and min_size are flexibly setting in this way. For the OOPS model (one occurrence of motif instance per sequence), we take max_size =* t* and min_size = 3*t*/4; for the ZOOPS model (zero- or one-motif occurrences per dataset sequence), the number of motif instances is less than the number of sequences and we take max_size = *t* and min_size =* t*/2; for TCM (two-component mixture) model, there are zero or more nonoverlapping occurrences. Generally, we take max_size = 3*t*/2 and min_size = *t*.

We first use six real DNA datasets to test the performance of our algorithm, including CREB, CRP, MEF2, MYOD, SRF, and TBP [26–28]. These datasets contain the sequences of different species, in which motif length varies from 6 to 18 and the number of motif instances is from 17 to 95. Note that, in CREM and CPR datasets, some sequences have two motifs, and in MYOD and SRF datasets, the number of motifs is more than two in some sequences. Using these datasets to test, we can check the performance and stability of our algorithm in different species. And the site information tagged in the dataset can help us have a better performance analysis and compare with other algorithms. The information of the six datasets is shown in Table 1.

Where *t* represents the sequence number, *n* is the sequence length, *l* is the motif length, *z* is the number of motif instances in the dataset, and *z*_avg is the average number of motif instances in each sequence.

We compare EPP algorithm with the widely used algorithms, MEME [10], GAME [29], VINE [14], and APMotif [15]. In order to achieve a fair comparison, we use the same motif length for each dataset and use the prior information as less as possible. We choose groups of different initiate sites for multirunning MEME because of the sensitive with initiate conditions. For the genetic-based algorithm GAME, the results are influence by the random seeds; thus, we run the algorithm 20 times and take the average. In each run, the search quantity of motif sets of GAME is 3 × 10^7^.

In order to evaluate the performance of the algorithms, we employ an evaluation method mixing the nucleotide level and the site level [30]. That is, if the predict sites and the real sites are shifting in three bases, it is a true instance. We employ three measures, Precision, Recall, and *F* score [31], which are defined as follows:

(18) Precision=correct  motifmotif  found,Recall=correct  motifture  found,F  score=2×Precision×RecallPrecision+Recall.

Here, Precision represents the probability of predicted instances which is influenced by false positive instances. Recall represents the probability of true positive instances. And *F* score is a measure which makes a balance between Precision and Recall, which reduces the influence of false positive. A high *F* score means the algorithm has good performance in both Precision and Recall.

**Figure 3:**
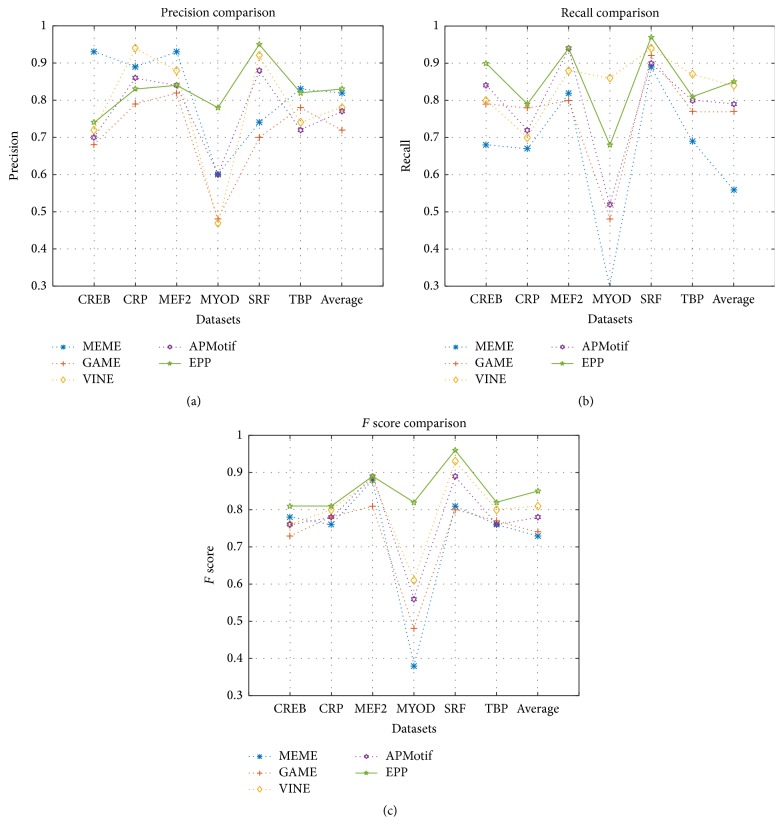
The accuracy comparison of MEME, GAME, VINE, APMotif, and EPP. (a) Precision comparison. (b) Recall comparison. (c) *F* score comparison.

Table 2 shows the results of MEME, GAME, VINE, APMotif, and EPP. It can be seen that EPP has a good performance of Precision on MYOD (0.78) and SRP (0.95). MEME has a high Precision on CREB (0.93), MEF2 (0.93), and TBP (0.83). VINE has a high Precision on CRP (0.94). In the respect of Recall, our algorithm performs well on CREB (0.90), CRP (0.79), MEF2 (0.94), and SFR (0.97). APMotif has the same Recall (0.94) on MEF2 with EPP. And VINE performs well on MYOD (0.86) and TBP (0.87). On the aspect of Precision and Recall, we can see that EPP has relatively small influence by the background. In the predicted instances, the true motif instances occupy a larger proportion. So on the aspect of *F* score, our algorithm has the best performance among the five algorithms; only APMotif has the same value on MEF2. The comparison of Precision, Recall, and *F* score is shown in Figure 3; we can find EPP has a stable performance on the average and performs well than the current widely used motif finding algorithms.

Table 3 shows the amount of subsets and the *l*-mers in each step, including the total *l*-mers, the thresholds of min_size and max_size, the amount of candidate subsets and qualified subsets, the *l*-mers in the qualified subsets, and the reducing number of *l*-mers. We can see that the our algorithm eliminates most of the candidate subsets by the projection step and the filter step; only dozens of subsets need to be refined. Meanwhile, the amount of *l*-mers has a great reduction, which is more than 90%. Such as TBP dataset, the amount of *l*-mers reduces by 99% and only two subsets need the refinement.

The running times of the datasets testing above are shown in Table 4. We implement EPP and APMotif in MATLAB under Windows. GAME is implemented in C under Linux. MEME and VINE are implemented through the website version. It is unfair to compare these algorithms implemented in different software, especially compared with website version. But the running time can explain that our algorithm can find the motifs in a reasonable and acceptable time. We report the computational time in the same experiment environment (2.67 G Hz CPU and 4 G memory). From Table 4, we can see that GAME and APMotif are obviously slower than EPP. The web version MEME and VINE are faster than EPP for most datasets. However, MEME needs to run several times for the different start points and VINE is a heuristic algorithm which will be slow with the data size increasing. EPP has the best time efficiency for TBP data because of the reduction of 99% redundant information.

**Figure 4:**
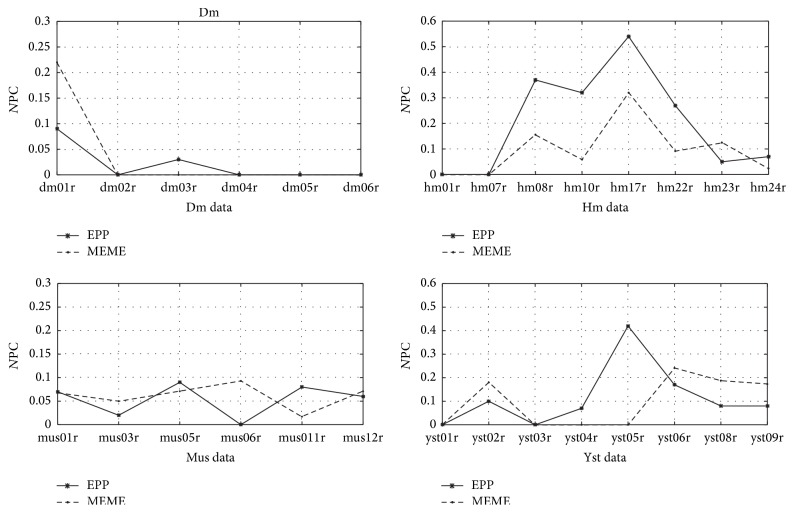
Results of EPP and MEME on Tompa datasets.

Besides the six real DNA datasets, we also use the Tompa data to test our algorithm. Tompa data is a standard data for evaluating new design motif finding method, including three types of data: Real, Generic, and Markov. Here, we select Real data which contains 52 groups of real promoter sequences extracted from TRANSFAC database and involves four species:* Drosophila melanogaster* (dm), Mouse (mus), Human (hm), and* Saccharomyces cerevisiae* (yst). It should be noted that some datasets of Tompa only have one sequence, such as dm02r and dm06r. Not each sequence contains the motif, such as dm01r, hm06r, hm11r, mus07r, and yst01r. And for most of the Tompa datasets, each sequence contains more than one motif, like hm08r, hm10r, mus11r, yst03r, and yst05r. Motifs are difficult to identify for the weak conservation in Tompa data. Thus, we select a part of the datasets to test, which are dm01r, dm02r, dm03r, dm04r, dm05r, and dm06r in Dm species; mus01r, mus03r, mus05r, mus06r, mus11r, and mus12r in Mus species; hm01r, hm07r, hm08r, hm10r, hm17r, hm22r, hm23r, and hm24r in Hm species; yst01r, yst02r, yst03r, yst04r, yst05r, yst06r, yst08r, and yst09r in Yst species (Figure 4). We use the measure based on the nucleotide level to evaluate the performance, because the number of motifs and the length of motifs are different in each sequence.

(19) $$\begin{array}{c} NPC=\frac{nTP}{\left(nTP+nFP+nFN\right)}, \end{array}$$

where *n*TP (true positive) represents the real sites in the predicted sites; *n*FP (false positive) are the fake sites in the predicted sites; *n*FN (false negative) represents the fake sites that do not predict. We also choose MEME as the reference algorithm to compare the performances. The length of motif ranges from 6 to 30 bps, and we output the best result. Figure 3 is the results of EPP and MEME. We can see that both EPP and MEME are hard to find the motifs in the one sequence data sets, such as dm02r and dm06r. For the datasets dm03r, dm04r, and dm05r, some sequences have several motifs but some sequences have no motif; for example, the third sequence of dm05r contains 9 motifs. This motif distribution makes it difficult to identify. Thus, both EPP and MEME have poor effect for the Dm spices. For the Hm spices, one notable feature is that the length of motifs changes a lot; for example, the motifs of hm01r range from 7 to 56 bps. We use the fixed motif length as before which can only predict a part of segment overlapping with the true motif. However, for the data motif length changing relatively small, like hm17r (10–17 bps), both EPP and MEME have the best results. And EPP has a higher accuracy than MEME in the Hm spices. For the Mus and the Yst data, most of the datasets contain less than 10 sequences (expect mus11r, yst03r, yst08r, and yst09r), and most of the sequences have multiple motifs of different lengths. From the experiment results, we find that EPP and MEME have their own advantages for these two species.

Through the experiments above, we can see the existing algorithms have poor performance on Topma data [32]. However, the different algorithms can complement and reinforce each other. For example, for the data mus06r, yst05r, and hm10r, EPP can have an effective prediction but the accuracy of MEME is worse. In recent research, the algorithm like Ensemble which merges the results of different algorithms can improve the accuracy effectively [33]. Moreover, the same results of the different algorithms can also enhance the prediction.

In order to show the effect of our algorithm, we also test the synthetic datasets which contain the low and high conservation positions. The synthetic datasets are generated under the following six combinations of three perspectives: (1) motif width: short (8–10 bp), middle (14–16 bp), and long (19–21 bp); (2) sequence length: 600 and number of sequences: 20; (3) motif conservation: low and high. For each combination, we sample 10 datasets which are generated randomly and embedded with the instances of a random motif. Specifically, in the high conservation aspect, the dominant nucleotide is generated with 0.91 probability on each position of the motif instance (while all other three nucleotides are generated with 0.03 each). In the low conservation aspect, only 60 percent of the positions in the motif instances are as highly conserved as those in the previous high conservation aspect, while the rest 40 percent of the positions are lowly conserved, where the dominant nucleotide is generated only with probability 0.55 (while all other three nucleotides are generated with 0.15 each) in every instance.

Table 5 shows the performance coefficient (*N*PC) of MEME, VINE, and EPP. From the results, we can see that all these compared algorithms have good performance on the high conservation dataset. Among these compared algorithms, EPP has the best results on three high conservation datasets (0.98, 0.99, and 1), which are higher than the other three algorithms. For the low conservation datasets, EPP has the highest accuracies among these compared algorithms. However, when the width of motif is short, motif instances are hard to distinguish from the background sequences; the accuracies of all the compared algorithms are low.

Meanwhile, we also use 12 TFs in mouse embryonic stem cell ChIP-seq datasets to test our algorithm. ChIP-seq is a technique coupling chromatin immunoprecipitation experiment with high-throughput sequencing [34, 35], which provides dataset of one or two magnitudes larger than a typical motif discovery dataset and sequences with a high resolution. Therefore, the tradition motif finding algorithms are hard to solve ChIP-seq data for the huge calculation. In order to improve the efficiency of EPP, the original dataset is equally divided into halves: a training set and a testing set. We run the projection and filter steps on the training set to generate the qualified subsets, and then run the refine step to search the instances and construct longer motifs on the testing set.  Table 6 shows the results of 12 TFs in mES ChIP-seq datasets discovered by our algorithm with the motifs found by Chen et al. with Weeder [36]. It can be seen that EPP is able to find the motif similar to the published one.

Chen et al. report a single motif with Weeder. Besides these primary motifs, our algorithm can find multiple motifs for each TF using the same datasets. For instance, Oct4 and Sox2 often form a heterodimer that binds a Oct4 motif located adjacent to a Sox2 motif, called the Sox-Oct motif [37]. In Sox2 and Oct4 dataset, EPP predicts not only the Sox-Oct composite motif bound by Sox2 and Oct4 complex but also the monomer motifs Sox2 (CCATTGTT) and Oct4 (TATGCAAAT). As discussed by Chen et al., Smad1 and Nanog frequently bind the same regions as Oct4 and Sox2, which raises a particular difficulty for motif discovery [38]. In Smad1 dataset, our algorithm finds motif “CCTTTGTC,” which matches a Sox2 motif and demonstrates the frequent cobinding relationship of Smad1 and Sox2 TFs. Furthermore, our algorithm was able to find the Nanog motif “CCATCAA,” which corresponds to an experimentally validated alternative Nanog motif [39].

In summary, EPP is a competitive algorithm to deal with motif discovery problem; our method has the following advantages: (1) the projection which deals with all the substrings does not miss any information in the data. That is, this step guarantees each substring may exist in a candidate subset. (2) The goal of finding motif is to find the substrings having the maximum IC, and the process of selecting the projection positon is also a part of maximizing IC. (3) The size of candidate subsets depends on the thresholds [min_size, max_size]. If a candidate subset is too large, it will contain too much background information. We continue to divide it; if a candidate subset is too small, the substring in it may be not enough to represent an effective motif. We abandon it. In some cases, motif instance may exist in the abandoned subset, but it still can make up by other subsets containing the motif instance. In the worst case, the number of the candidate subsets is* n*/min_size, where* n* is the number of all substrings. However, in practice, this number will drastically reduce. The number of candidate subsets may be only a few hundred for 10^6^ substrings. (4) There are often some meaningless DNA segments in real data, such as duplicate “AAAAAAAAAA” or “CGCGCGCGCG.” These segments will generate the same duplicate substrings which cause redundant computation. Through the projection step of our algorithm, these segments will be very easy to find and discard.

In addition, the computation complexity of EPP mainly depends on projection step and refinement step. Suppose the time of projection is *h*, in each projection, the computation complexity of calculating relative entropy is O(*nl*); then, the computation complexity of the projection step is O(*hnl*). Since the order of magnitude of *h* and *l* is 10, and *n* is usually less than 10^6^, the order of magnitude of projection is about 10^8^. In the refinement, the number of qualified subsets is about 10^2^ for 10^6^ substrings. the computation complexity of refinement in each qualified subset is O(*nl*). So the order of magnitude of refinement is 10^8^ which is totally acceptable.

## 4. Conclusions

We propose a new probability algorithm named EPP for identifying motifs in DNA datasets. EPP presents a new entropy-based position projection to divide original dataset and remove a large amount of redundant information. Experimental results show that EPP is able to efficiently and effectively identify motifs in DNA sequences and ChIP-seq datasets. However, the functions of some motifs are still unknown; the analysis of motifs in these complex transcriptional regions is needed. In addition, with the increase of data size, designing the parallel algorithm to handle big data is also a key issue for the future study.

## Figures and Tables

**Algorithm 1:**
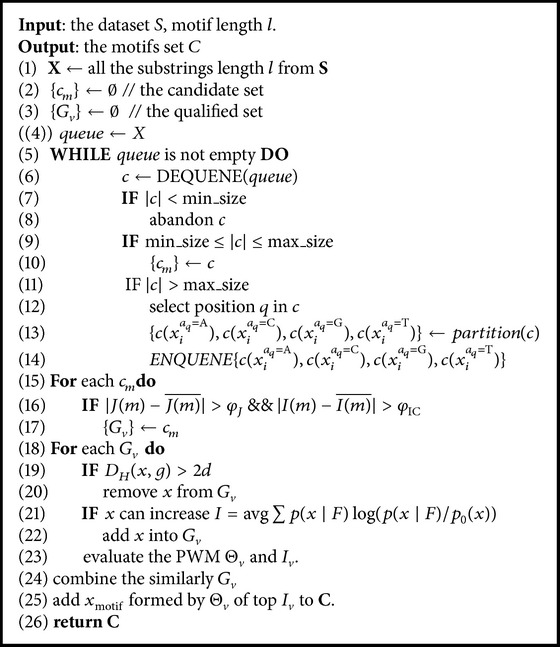


**Table 1:** The information of six DNA datasets.

Datasets	*t*	*n*	*l*	*z*	*z_*avg
CREB	17	200	8	19	1.12
CRP	18	105	18	23	1.28
MEF2	17	200	10	17	1
MYOD	17	200	6	21	1.23
SRF	20	200	10	36	1.8
TBP	95	200	7	95	1

**Table 2:** The comparison of MEME, GAME, VINE, APMotif, and EPP on six DNA datasets.

Datasets	MEME			GAME			VINE			APMotif			EPP		
	*P*	*R*	*F*	*P*	*R*	*F*	*P*	*R*	*F*	*P*	*R*	*F*	*P*	*R*	*F*
CREB	**0.93**	0.68	0.78	0.68	0.79	0.73	0.72	0.80	0.76	0.70	0.84	0.76	0.74	**0.90**	**0.81**
CRP	0.89	0.67	0.76	0.79	0.78	0.78	**0.94**	0.70	0.80	0.86	0.72	0.78	0.83	**0.79**	**0.81**
MEF2	**0.93**	0.82	0.88	0.82	0.80	0.81	0.88	0.88	0.88	0.84	**0.94**	**0.89**	0.84	**0.94**	**0.89**
MYOD	0.60	0.28	0.38	0.48	0.48	0.48	0.47	**0.86**	0.61	0.60	0.52	0.56	**0.78**	0.68	**0.82**
SRF	0.74	0.89	0.81	0.70	0.92	0.80	0.92	0.94	0.93	0.88	0.90	0.89	**0.95**	**0.97**	**0.96**
TBP	**0.83**	0.69	0.76	0.78	0.77	0.77	0.74	**0.87**	0.80	0.72	0.80	0.76	0.82	0.81	**0.82**

Average	0.82	0.56	0.73	0.72	0.77	0.74	0.78	0.84	0.81	0.77	0.79	0.78	**0.83**	**0.85**	**0.85**

**Table 3:** The subsets and *l-*mers amount of EPP.

Datasets	Total *l-*mers	[min_size, max_size]	The number of candidate subsets	The number of qualified subsets	The *l-*mers in qualified subsets	Reducing amount of *l-*mers
CREB	3294	[15,19]	66	4	65	98%
CRP	1584	[16,24]	31	5	104	98%
MEF2	3247	[9,17]	176	33	335	90%
MYOD	3315	[17,23]	55	6	111	97%
SRF	3820	[20,30]	73	13	310	92%
TBP	18430	[80,95]	32	2	175	99%

**Table 4:** The computational time comparison.

Datasets	MEME	GAME	VINE	APMotif	EPP
CREB	1.52	134.00	4.82	71.23	17.52
CRP	0.60	391.04	2.61	97.04	8.91
MEF2	2.01	113.25	7.37	135.83	21.91
MYOD	2.25	96.08	8.25	68.36	30.27
SRF	2.12	223.56	10.11	147.29	28.28
TBP	39.05	786.32	55.53	280.43	10.83

**Table 5:** The performance coefficient of MEME, VINE, and EPP on the synthetic datasets.

Datasets		Algorithm		
Width	Con	MEME	VINE	EPP
Short	Low	0.32	0.24	0.32
Middle	Low	0.88	0.72	0.90
Long	Low	0.98	0.88	0.98
Short	High	0.91	0.96	0.98
Middle	High	0.98	0.99	0.99
Long	High	1	1	1

**Table 6:** Results of the mouse embryonic stem cell data.

Datasets	Length	Seq. #	EPP	Weeder
*CTCF*	11	39601	**CCA**GA**AG**A**GGGCG**	TNG**CCA**CC**AG**G**GGGCGCNN**
*cMyc*	9	3422	**GC**T**CGTGGC**	C**GC**A**CGTGGC**
*Esrrb*	11	21644	**GGTCAAGGTCA**	**GGTCAAGGTCA**
*Klf4*	10	10872	**GGGTGTGGCC**	**GGGTGTGGCC**
*Nanog*	7	10342	**CCATT**C**T**	**CCATT**G**T**CTNNN
*nMyc*	10	7181	**CGCACGTGGC**	**CGCACGTGGC**
*Smad1*	16	1126	**C**T**TTTGTTAT**T**CAAAT**	**C**C**TTTGTTAT**G**CAAAT**
*Oct4*	15	3775	**C**A**TTGTTATGCAAA**	**C**T**TTGTTATGCAAA**T
*STAT3*	9	2546	**TTCCTGGAA**	T**TTCCNGGAA**
*Sox2*	10	4525	**TTGTTATGCA**	CA**TTGTNATGCA**AAT
*Tcfcp2l1*	11	26907	**C**CAGC**C**T**A**G**CC**	**C**CGGTT**C**A**A**A**CC**GG
*Zfx*	10	10336	**CTAGGCCGCG**	CG**CNAGGCCGCG**
